# FITA-Containing 2,4-Dinitrophenyl Alkylthioether-Based Probe for Detection and Imaging of GSH

**DOI:** 10.3390/s25010034

**Published:** 2024-12-24

**Authors:** Yalun Dong, Liyue Wang, Wenfang Liang, Jiqin Zhu, Lu Sun, Long Yi

**Affiliations:** 1 State Key Laboratory of Organic-Inorganic Composites and Beijing Key Lab of Bioprocess, Beijing University of Chemical Technology, Beijing 100029, China; 2022400048@mail.buct.edu.cn (Y.D.); m13593008045@163.com (W.L.); 2 Tianjin Key Laboratory on Technologies Enabling Development of Clinical Therapeutics and Diagnostics, School of Pharmacy, Tianjin Medical University, Tianjin 300070, China; wly13034198045@163.com (L.W.); sunlu@tmu.edu.cn (L.S.)

**Keywords:** GSH, 2,4-dinitrophenyl alkylthioether, FITA, fluorescent probe

## Abstract

Glutathione (GSH) plays a crucial role in various physiological processes and its imbalances are closely related to various pathological conditions. Probes for detection and imaging of GSH are not only useful for understanding GSH chemical biology but are also important for exploring potential theranostic agents. Herein, we report a fast intramolecular thiol-activated arylselenoamides (**FITA**)-based fluorescent probe using 2,4-dinitrophenyl alkylthioether as a sulfydryl-selective receptor for the first time. The fluorescence of the probe was low due to the double effects of PET, while the probe exhibits an 86-fold fluorescence enhancement at 460 nm after GSH activation and a detection limit of 0.95 μM. Furthermore, the probe is low-toxic and capable of imaging cellular GSH. This work further expands the design and applicability of the **FITA**-based platform, offering a new thiol-deprotection strategy for development of fluorescent probes.

## 1. Introduction

Biothiols play distinct but crucial roles in maintaining intracellular redox homeostasis and protecting the cells from oxidative stress [1,2,3,4,5]. In particular, the most abundant cellular biothiol, glutathione (GSH), acts as a central role due to its transformation between sulfhydryl reduced form (GSH) and disulfide oxidized form (GSSG) [6]. Abnormal levels of these closely related small-molecule biothiols are associated with many diseases, including liver damage, AIDS, cancer, Alzheimer’s disease, and aging [7,8,9,10,11,12]. In addition, recent studies suggest that tumor cells contain up to millimolar concentrations of endogenous GSH, and excess GSH can shelter the cancer cells from radiation therapy or chemotherapy [13,14]. To this end, detection and elimination of GSH is conducive for cancer diagnosis and therapy [15,16,17,18,19,20,21,22]. Due to its biological and clinical importance, the development of chemical tools for GSH determination is of great importance.

**Scheme 1:**
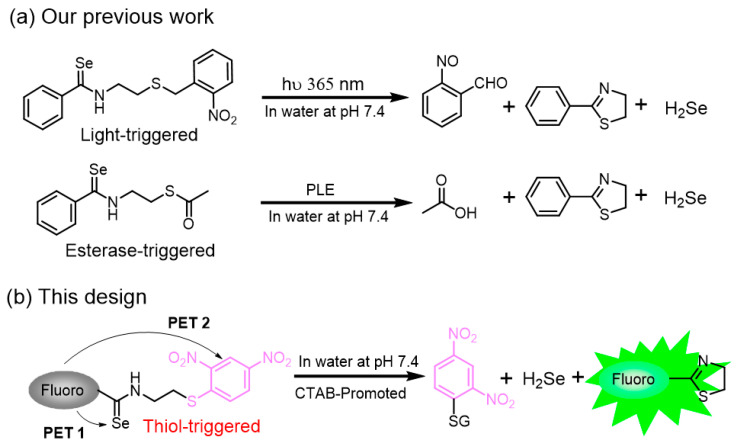
(**a**) Reported photo- and esterase-triggered **FITA**-based platforms. (**b**) A new fluorogenic **FITA**-based platform that contains 2,4-dinitrophenyl alkylthioether as a new thiol-selective receptor.

The high sensitivity, excellent selectivity, and non-invasive properties make small-molecule fluorescent probes stand out in bioanalytical fields when compared with other analytical methods [22,23,24,25,26,27,28]. In the past decade, 2,4-dinitrophenyl (DNB) ether motifs and its derivatives, such as 2,4-dinitrobenenesulfonate motifs and DNB aryl-thioether motifs, have been used broadly to generate fluorescent probes for reactive sulfhydryl species [9,29,30,31,32,33,34,35,36,37,38,39,40,41,42,43,44]. These reported probes (Figure S1) share a homologous sensing mechanism: the substitution of 2- and 4-positions on the DNB’s aromatic group by strong electron-withdrawing nitro groups makes the 1-position carbon activated for S_N_Ar attack by thiol-based nucleophiles. Moreover, these DNB-based probes have excellent properties and good biocompatibility, which make them suitable tools for imaging biothiols in living cells. Despite these advancements, biocompatible *C*-*S* bond cleavage-based receptors are rarely reported [45,46]. Given these, we envisioned that the DNB alkylthioether might serve as a new receptor for thiol detection and imaging. On the other hand, we recently reported a new strategy for H_2_Se donors and fluorescent probes development based on the fast intramolecular thiol-activated arylselenoamides (**FITA**) [47]. In this work, we combined the **FITA** platform and the DNB alkylthioether to develop a new fluorescent probe (Scheme 1), which was successfully applied to detect GSH in buffers and in cells.

## 2. Materials and Methods

### 2.1. Materials

6-(Dimethylamino)-2-naphthoic acid and *S*-tritylcysteamine hydrochloride were obtained from Bide Pharmaceutical Technology Co., Ltd. (Shanghai, China). *N*-(3-Dimethylaminopropyl)-*N*′-ethylcarbodiimide hydrochloride (EDC), 4-dimethylaminopyridine (DMAP), triethylsilane (TES), 2,4-dinitrofluorobenzene (DNFB), hydrogen peroxide solution (3w% H_2_O_2_ in H_2_O), and cysteine (Cys) were obtained from Shanghai Macklin Biochemical Technology Co., Ltd. (Shanghai, China). Trifluoroacetic acid (TFA) and sodium sulfide (Na_2_S) were obtained from Shanghai Aladdin Biochemical Technology Co., Ltd. (Shanghai, China). Triethylamine (TEA), sodium sulfite (Na_2_SO_3_), sodium bisulfite (NaHSO_3_), and sodium sulfate (Na_2_SO_3_) were obtained from Tianjin Fuchen Chemical Reagents Co., Ltd. (Tianjin, China). Woollins’ reagent and *N*-ethylmaleimide (NEM) were obtained from Shanghai Adamasi Reagents Co., Ltd. (Shanghai, China). GSH and homocysteine (Hcy) were obtained from TCI (Shanghai, China). Sodium polysulfide was obtained from Chengdu Zero Six Biotechnology Co., Ltd. (www.ix-r.com, Chengdu, China). Hexadecyl trimethyl ammonium bromide (CTAB) was obtained from Sinopharm Chemical Reagent Co., Ltd. (Shanghai, China).

All chemicals and solvents used for synthesis were purchased from commercial suppliers and applied directly in the experiments without further purification. The progress of the reactions was monitored via TLC on precoated silica plates, and spots were visualized via UV light or iodine. Merck silica gel 60 (70–200 mesh) was used for general column chromatography purification. ^1^H, ^13^C{^1^H} NMR spectra were recorded on a Bruker 400 (400 MHz for ^1^H NMR, 101 MHz for ^13^C NMR), and ^77^Se{^1^H} NMR was recorded on a Bruker 600 (114 MHz for ^77^Se NMR) Nuclear Magnetic Resonance Spectrometer (Bruker, Madison, WI, USA). Chemical shifts are reported in parts per million relative to internal standard tetramethylsilane (Si(CH_3_)_4_ = 0.00 ppm) or residual solvent peaks (CDCl_3_ = 7.26 ppm; DMSO-*d*_6_ = 2.50 ppm). High-resolution mass spectra (HRMS) were recorded on an Agilent 6540 UHD Accurate-Mass Q-TOFLC/MS (Agilent Technologies Inc., Santa Clara, CA, USA) with positive and negative ion modes. The UV-visible spectra were recorded on a UV-6000 UV-VIS-NIR-spectrophotometer (METASH, Shanghai, China). Fluorescence studies were performed using F-280 spectrophotometer (Tianjin Gangdong Sci & Tech., Development Co., Ltd., Tianjin, China). Cellular bioimaging was carried out on a confocal microscope (Olympus FV1000, Olympus Corporation, Tokyo, Japan).

### 2.2. Synthesis of Probe (Scheme 2)

A mixture of 6-(dimethylamino)-2-naphthoic acid (860 mg, 4 mmol), EDC (1.92 g, 10 mmol), and DMAP (61 mg) in THF (50 mL) was stirred at 0 °C for 10 min, and then *S*-tritylcysteamine hydrochloride (2.5 g, 7 mmol) and TEA (718 mg, 7 mmol) in 10 mL THF were added slowly. The resultant solution was stirred for 12 h at room temperature. The solvent was removed under reduced pressure, and the residue was redissolved with CH_2_Cl_2_, which was washed with water and brine, dried over Na_2_SO_4_, and concentrated under reduced pressure. The resultant crude residue was purified via silica gel column chromatography with CH_2_Cl_2_/MeOH (100/0.5) to give **1** as a yellow solid (1.9 g, yield 92%). ^1^H NMR (400 MHz, CDCl_3_) *δ* 8.11 (s, 1H), 7.76 (d, *J* = 9.1 Hz, 1H), 7.68 (dd, *J* = 8.6, 1.7 Hz, 1H), 7.66–7.62 (m, 1H), 7.47–7.42 (m, 6H), 7.30–7.25 (m, 6H), 7.23–7.15 (m, 4H), 6.89 (d, *J* = 2.4 Hz, 1H), 6.47–6.42 (m, 1H), 3.37 (dd, *J* = 12.3, 6.1 Hz, 2H), 3.08 (s, 6H), 2.57 (t, *J* = 6.3 Hz, 2H). ^13^C NMR (101 MHz, CDCl_3_) *δ* 167.7, 149.7, 144.8, 136.7, 130.0, 129.6, 128.7, 128.1, 127.5, 126.9, 126.3, 125.5, 124.1, 116.6, 105.5, 66.9, 40.6, 38.7, 32.5. HRMS (ESI): *m*/*z* [M + H]^+^ calcd. for C_34_H_33_N_2_OS^+^: 517.2308; found: 517.2324.

To a solution of **1** (1.03 g, 2 mmol) in CH_2_Cl_2_ (16 mL), TFA (4 mL) and TES (2 mL) were added. After stirring for 30 min at room temperature, the solvent was removed under reduced pressure, and the crude residue was purified via silica gel column chromatography with CH_2_Cl_2_/MeOH (100/3) to give a light-yellow solid, which was redissolved in CH_2_Cl_2_ (20 mL) under argon gas protection. Then, DNFB (750 mg, 4 mmol) and TEA (610 mg, 6 mmol) were added. After stirring at room temperature overnight, the reaction solution was diluted with CH_2_Cl_2_ and then washed with water and brine, dried over Na_2_SO_4_, and concentrated under reduced pressure. The crude residue was purified via silica gel column chromatography with CH_2_Cl_2_/MeOH (100/0.5) to give **2** as a red solid (850 mg, yield 96%). ^1^H NMR (400 MHz, DMSO-*d*_6_) *δ* 8.85 (d, *J* = 2.5 Hz, 1H), 8.78 (t, *J* = 5.5 Hz, 1H), 8.41 (dd, *J* = 9.0, 2.5 Hz, 1H), 8.22 (s, 1H), 8.09 (d, *J* = 9.1 Hz, 1H), 7.79 (d, *J* = 9.1 Hz, 1H), 7.75 (dd, *J* = 8.7, 1.7 Hz, 1H), 7.67 (d, *J* = 8.7 Hz, 1H), 7.27 (dd, *J* = 9.1, 2.5 Hz, 1H), 6.94 (d, *J* = 2.3 Hz, 1H), 3.62 (dd, *J* = 12.5, 6.4 Hz, 2H), 3.44 (t, *J* = 6.7 Hz, 2H), 3.04 (s, 6H). ^13^C NMR (101 MHz, DMSO-*d*_6_) *δ* 166.8, 149.5, 145.3, 144.9, 143.6, 136.2, 129.7, 128.3, 127.3, 126.7, 125.6, 124.7, 124.2, 121.3, 116.6, 105.0, 40.0, 37.7, 31.3. HRMS (ESI): *m*/*z* [M + H]^+^ calcd. for C_21_H_21_N_4_O_5_S^+^: 441.1227; found: 441.1232.

A mixture of **2** (220 mg, 0.5 mmol) and Woollins’ reagent (266 mg, 0.5 mmol) in dried toluene (15 mL) was stirred at 110 °C in a sealed tube for 2 h. Then, the solvent was removed under reduced pressure, and the crude residue was purified via silica gel flash column chromatography (CH_2_Cl_2_) to give **FITA-FD3** as a red solid (16 mg, yield 6%). ^1^H NMR (400 MHz, DMSO-*d*_6_) *δ* 10.96 (t, *J* = 5.1 Hz, 1H), 8.87 (d, *J* = 2.5 Hz, 1H), 8.43 (dd, *J* = 9.0, 2.5 Hz, 1H), 8.14 (d, *J* = 9.1 Hz, 1H), 8.12 (s, 1H), 7.86–7.81 (m, 1H), 7.81–7.79 (m, 1H), 7.62 (d, *J* = 8.8 Hz, 1H), 7.27 (dd, *J* = 9.1, 2.4 Hz, 1H), 6.93 (d, *J* = 1.9 Hz, 1H), 4.15 (dd, *J* = 12.3, 6.4 Hz, 2H), 3.69 (t, *J* = 6.7 Hz, 2H), 3.05 (s, 6H). ^13^C NMR (101 MHz, DMSO-*d*_6_) *δ* 202.0, 149.5, 145.0, 144.8, 143.8, 136.1, 136.0, 130.0, 128.5, 127.4, 126.6, 125.8, 125.2, 124.4, 121.4, 116.7, 104.9, 47.3, 40.2, 29.3. ^77^Se NMR (114 MHz, DMSO-*d*_6_) *δ* 559.6. HRMS (ESI): *m*/*z* [M + H]^+^ calcd. for C_21_H_21_N_4_O_4_SSe^+^: 505.0443; found: 505.0432.

### 2.3. Mechanism Verification

With the probe **FITA-FD3** in hand, we first analyzed the reaction of **FITA-FD3** and GSH by HRMS. Compound **FITA-FD3** (0.5 mM) in PBS buffer (50 mM, pH 7.4, containing 1 mM CTAB) was incubated with GSH (5 mM) for 1 h at room temperature. Then, the reaction solution was checked by HRMS tests at both positive and negative ion modes.

### 2.4. Spectra Tests and Reaction Kinetics

All spectroscopic measurements were performed in degassed phosphate buffer (PBS, 50 mM, pH 7.4, containing 1 mM CTAB). Probe **FITA-FD3** was dissolved in DMSO to prepare a stock solution of 10 mM. Each reaction mixture was shaken uniformly before spectra measurement. All measurements were performed in a 3 mL corvette with 2 mL solution at room temperature, and all fluorescence spectra were obtained via excitation at 350 nm with slit width 5/5 nm. pH-dependent (2.0, 3.0, 4.0, 5.5, 6.5, 7.4, 9.0) fluorescence spectra were also recorded.

Probe **FITA-FD3** (5 μM) was incubated with or without GSH (1–5 mM from 50 mM stock solution) in PBS (pH 7.4, 1 mM CTAB) at room temperature. Time-dependent fluorescence spectra were recorded, and emission intensities at 460 nm were analyzed for kinetic studies.

### 2.5. Titration Experiments

A mixture of 1 μL of the probe (final 5 μM) and different concentrations of GSH in 2 mL PBS was integrated and thoroughly mixed. After 1 h of incubation, the fluorescence spectra were recorded and emission intensities at 460 nm versus GSH concentrations were used to obtain the titration curve. The detection limit (LOD) was calculated by the 3σ/*k* method [24], where σ is the standard deviation of fluorescence intensity of only **FITA-FD3** in buffer, and *k* is the slope between the fluorescence intensities and GSH concentrations.

### 2.6. Selective Tests

The selectivity was measured by fluorescence responses (λ_em_ = 460 nm) of **FITA-FD3** (5 μM) with various species in the absence or presence of GSH for 1 h of incubation. All analytes were prepared as stock solutions in degassed water (100 mM Na_2_S, Hcy, Na_2_S_4_, Na_2_SO_3_, NaHSO_3_, Cys, Na_2_SO_4_; 3w% H_2_O_2_, 50 mM GSH). All measurements were performed in triplicates in a 3 mL sealed cuvette with 2 mL of solution using the same parameters as in Section 2.4.

### 2.7. Cytotoxicity and Cell Imaging

Cytotoxicity: HeLa (human cervical cancer) cells were seeded and cultured based on our previous methods [48]. The cytotoxicity of probe **FITA-FD3** was determined via Cell Counting Kit-8 (CCK-8) assay. Briefly, HeLa cells were seeded into a 96-well plate and cultured for 24 h before experiments. After that, the culture medium was replaced with a fresh one, and the cells were incubated with different concentrations of probe **FITA-FD3** (0, 5, 10, 25, and 50 μM) for 24 h. Then, the culture medium was replaced with 100 μL DMEM containing 10% (*v*/*v*) CCK-8 reagent, and the plate was incubated for 1 h. Finally, the absorbance intensity in each well was detected at 450 nm by a microplate spectrophotometer (Thermo Multiskan Go, Thermo Fisher Scientific Oy, Vantaa, Finland).

Imaging in cells: The feasibility of probe **FITA-FD3** to detect intracellular GSH was evaluated via fluorescence imaging. In the experimental group, cells were co-incubated with probe **FITA-FD3** (5 μM) and CTAB (100 μM) for 30 min, while the negative control group cells were pre-treated with a thiol blocking reagent NEM (1 mM) for 30 min and then incubated with probe **FITA-FD3** (5 μM) and CTAB (100 μM) for 30 min. Moreover, the positive control group cells were pre-treated with NEM (1 mM) for 30 min, and then the culture medium was replaced with fresh one, and the cells were incubated with probe **FITA-FD3** (5 μM), CTAB (100 μM), and GSH (3 mM) for another 30 min. After these incubations, the cells were quickly washed with PBS and then fixed with 4% paraformaldehyde solution for 15 min. After that, the cells were washed with PBS and imaged using a confocal microscope (Olympus FV1000) with a 40 × objective lens. The emission was collected at the channel (425–525 nm) with 405 nm excitation [48].

## 3. Results

### 3.1. Reaction Mechanism Verification

In our previous work, we identified that both light- and esterase-activated thiol can rapidly activate an intramolecular arylselenoamide at pH 7.4 (*t*_1/2_ < 1 min) to remove the selenium quenching moiety (Scheme 1) [47]. Then, we hoped to further expand the application scope of this **FITA** platform and find a new sulfhydryl-protective group that could be triggered by various stimuli. Herein, we rationally designed a new fluorescent probe **FITA-FD3** that contains DNB alkylthioether as a new thiol receptor.

**Figure 1:**
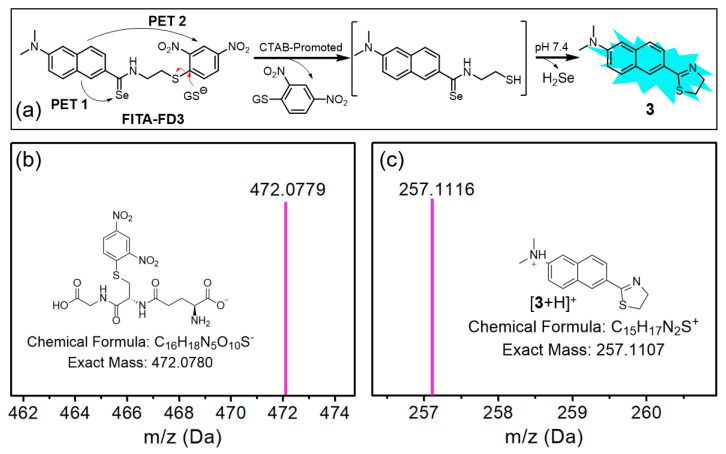
(**a**) The proposed sensing mechanism of **FITA-FD3** and GSH. (**b**,**c**) HRMS results obtained from the text in Section 2.3, supporting the proposed mechanism.

The structures of synthetic compounds were confirmed by ^1^H, ^13^C{^1^H}, and ^77^Se{^1^H} NMR spectroscopy, and HRMS (Figures S2–S11). Based on the documented low solubility of DNB-containing compounds, we used cetyltrimethylammonium bromide (CTAB, 1.0 mM) to increase the solubility and fluorescent response of **FITA-FD3** for the following measurements. In addition, GSH was used as a representative thiol trigger in the tests. We proposed that the ionized sulfhydryl group in GSH could attack the DNB thioether of **FITA-FD3**, resulting in a thiolysis reaction to liberate a sulfhydryl group for **FITA**, as well as a byproduct **DNB-SG** (Figure 1a). As expected, the probe solution is non-fluorescence due to possible dual photoinduced electron transfer (PET) processes with selenium and DNB moieties, and a strong fluorescence of the probe solution could be observed after the GSH-activated reaction (Figure S12). In UV-vis spectra, little wavelength changes after reaction with GSH also support the PET sensing mechanism (Figure S13). The fluorescence of the GSH-activated **FITA-FD3** is significantly red-shifted when comparing with a 6-(dimethylamino)-2-naphthylamide derivative **1**, possibly due to the extended π conjugation of the fluorophore with dihydrothiazole in **3** (Figure S12). In addition, probe **FITA-FD3** exhibited significantly lower fluorescence response under mildly acidic conditions compared with nearly neutral conditions (Figure S14), supporting the nucleophilic attack of anionic GS^−^ toward the probe. It is noted that the emission wavelengths of **3** in CTAB-containing and CTAB-free buffers are 460 nm and 485 nm [47], respectively, suggesting the existence of intermolecular interactions in hydrophobic microenvironments for the intramolecular charge transfer (ICT)-based fluorophore. Moreover, the expected products **3** and **DNB-SG** were identified via HRMS (Figure 1b,c), and the releasing H_2_Se was also qualitatively confirmed (Figure S15). Taken together, the results support a reaction and sensing mechanism of the thiolysis of DNB in **FITA-FD3** to generate the fluorophore **3**.

### 3.2. Reaction Kinetics

**Figure 2:**
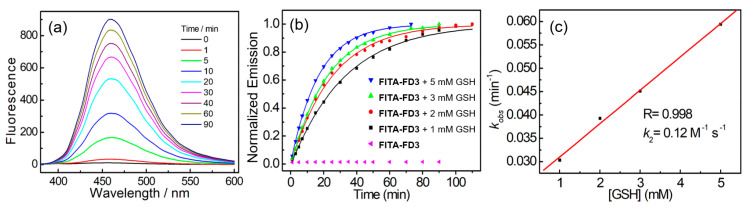
(**a**) Time-dependent fluorescence spectra of **FITA-FD3** (5 μM) upon treatment with GSH (3 mM) in PBS (50 mM, pH 7.4, containing 1 mM CTAB) at room temperature (excitation at 350 nm). (**b**) Time-dependent emissions at 460 nm of **FITA-FD3** with or without GSH. The solid line represents the best fit with a single-exponential function. (**c**) Linear relationship between the concentration of GSH and *k_obs_*. The slope of the best linear fit gives the reaction rate *k*_2_ (M^−1^ s^−1^).

In our subsequent investigation, we first delved into the fluorescence response of **FITA-FD3** to varying concentrations of GSH to quantify the kinetic rate (Figure 2). The probe is stable in PBS (50 mM, pH 7.4, containing 1 mM CTAB), and after reaction with GSH, about 86-fold turn-on at 460 nm was observed due to the formation of **3**. Multi-group time-dependent fluorescence intensities at 460 nm of **FITA-FD3** in the presence of different concentrations of GSH were recorded for kinetics studies (Figure S16). The pseudo-first-order rate *k_obs_* was determined by fitting the data with a single exponential function. The linear fit between *k_obs_* and the concentrations of GSH provided the reaction rate (*k*_2_) as 0.12 M^−1^ s^−1^, which was a moderate rate and beneficial for GSH probe selectivity.

### 3.3. Titration Experiments of **FITA-FD3**

**Figure 3:**
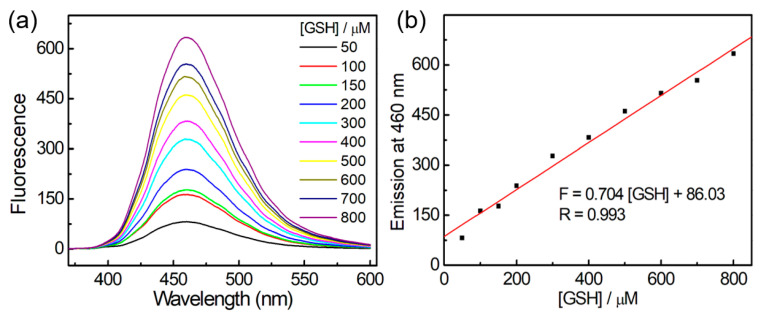
(**a**) Fluorescence spectra of titration curve of **FITA-FD3** (5 μM) towards GSH (50–800 μM) in PBS (pH 7.4, containing 1 mM CTAB); excitation: 350 nm. (**b**) The emission at 460 nm corresponding to (**a**). The solid line represents the best linear fit.

Encouraging with these results, we next investigated the concentration-dependent characteristics of probe **FITA-FD3**. As shown in Figure 3, the fluorescence intensities at 460 nm of the reaction solution exhibited a linear response to the GSH concentrations within the range of 50–800 µM. For determination of the standard deviation σ, ten samples of 5 µM probe **FITA-FD3** in 2 mL degassed PBS buffer (pH 7.4, containing 1 mM CTAB) were incubated for 1 h. Then, the fluorescence intensity at 460 nm of each sample was separately recorded (11.71, 11.87, 12.31, 12.10, 12.03, 12.24, 12.36, 12.37, 12.26, 12.27) for the determination of σ as 0.222. On the other hand, the linear fit between the emission at 460 nm and the concentration of GSH gave the slope *k* as 0.704. The calculated limit of detection (LOD) stands at 0.95 μmol/L. These results reveal that **FITA-FD3** displays good sensitivity to GSH, offering promising applications in various fields.

### 3.4. Selective Analysis Experiments

**Figure 4:**
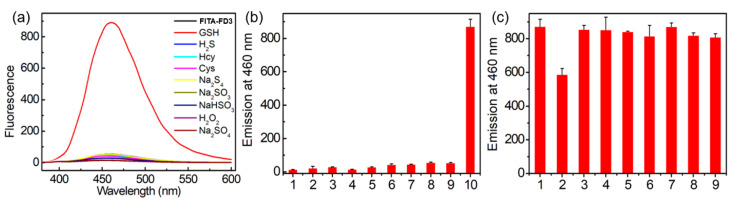
(**a**) Representative fluorescence spectra of **FITA-FD3** (5 µM) with various species in PBS (pH 7.4, containing 1 mM CTAB). The analytes contained 100 μM Na_2_S, Hcy, Na_2_S_4_, Na_2_SO_3_, NaHSO_3_, H_2_O_2_, or Na_2_SO_4_; 200 μM Cys; or 3 mM GSH. Excitation: 350 nm. (**b**) Emission at 460 nm of **FITA-FD3** (5 μM) with analytes at 25 °C for 1 h of incubation. Lane 1, only **FITA-FD3**; lanes 2–8, 100 μM H_2_O_2_, Na_2_SO_3_, Na_2_SO_4_, NaHSO_3_, Na_2_S_4_, Hcy, and Na_2_S, respectively; lane 9, 200 μM Cys; lane 10, 3 mM GSH. Excitation at 350 nm. (**c**) Emission at 460 nm of **FITA-FD3** (5 μM) toward various species in the presence of 3 mM GSH and other species at 25 °C for 1 h of incubation. Lane 1, only GSH; lanes 2–8, 100 μM H_2_O_2_, Na_2_SO_3_, Na_2_SO_4_, NaHSO_3_, Na_2_S_4_, Hcy, and Na_2_S, respectively; lane 9, 200 μM Cys. Data are expressed as mean ± S.D. (*N* = 3).

To examine the selectivity of probe **FITA-FD3** for biothiols, various biologically relevant species (H_2_S, Hcy, Cys, Na_2_S_4_, Na_2_SO_3_, NaHSO_3_, H_2_O_2_, Na_2_SO_4_) were used to incubate with probe **FITA-FD3** for 1 h in PBS buffer and their fluorescence response was measured separately. Considering the physiological concentrations of biothiols [14,24], we employed 100 μM for H_2_S and Hcy, 200 μM for Cys, and 3 mM for GSH in the tests. As expected, the fluorescence response of other tested molecules was far lower than that of GSH (Figure 4a,b). Further co-incubation analyses revealed that the fluorescence intensity was not affected by analytes, while the slightly less off–on response for H_2_O_2_ may be due to the direct reaction of H_2_O_2_ and GSH, resulting in reducing concentration of GSH during the activation of **FITA-FD3** (Figure 4c). In addition, GSH triggered the highest fluorescent off–on response of the probe compared with the same concentration of Cys, Hcy, or H_2_S (Figure S17), possibly because the two carboxyl groups in GSH enable its strong interaction with positively charged CTAB micelles containing the hydrophobic probe, thus causing higher local concentration and faster reaction rate of FITA-FD3 and GSH [39]. Taken together, probe **FITA-FD3** is selective toward GSH over other biologically relevant species.

### 3.5. Potential Applications in Cells

**Figure 5:**
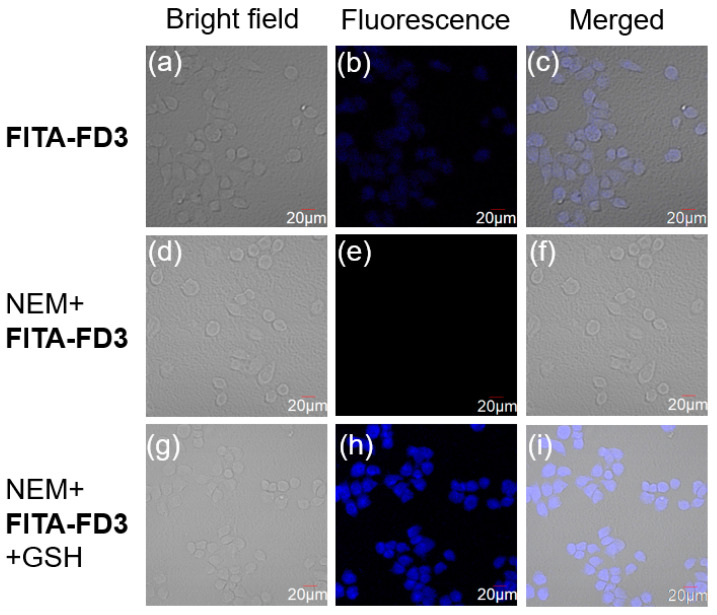
(**a**–**c**) Cells were incubated with **FITA-FD3** (5 µM) for 30 min. (**d**–**f**) Cells were pre-incubated with NEM (1 mM) for 30 min and then incubated with **FITA-FD3** (5 µM) for 30 min. (**g**–**i**) Cells were pre-incubated with NEM (1 mM) for 30 min and then incubated with **FITA-FD3** (5 µM) and GSH (3 mM) for 30 min. Bright-field (**a**,**d**,**g**), fluorescence channel (**b**,**e**,**h**), and merged (**c**,**f**,**i**). Scale bar: 20 μm.

Next, cytotoxicity of **FITA-FD3** was evaluated via CCK-8 assay. The results suggested that probe **FITA-FD3** almost had no obvious effect on HeLa cells’ viability when the probe concentration was under 5–50 μM after 24 h incubation (Figure S18), suggesting the good biocompatibility of probe **FITA-FD3**. Then, fluorescence imaging experiments were carried out to evaluate if the probe was suitable for imaging GSH in cells. As shown in Figure 5, when HeLa cells were incubated with probe **FITA-FD3** for 30 min, an obvious fluorescence was observed, and such optical signal of the probe should be “practically” reflecting the physiological levels of GSH in cells, because GSH occupies the majority of the biothiols in physiological samples. While in the negative control group, in which cells were pre-treated with the thiol blocking reagent *N*-ethylmaleimide (NEM, 1 mM) for 30 min, the fluorescence signal was very weak. Besides, the positive control group cells were pre-treated with NEM for 30 min then co-incubated with probe **FITA-FD3** and exogenous GSH for the same time; a strong fluorescence was observed. These results suggest that probe **FITA-FD3** is a satisfying tool for imaging both endogenous and exogenous GSH in cells.

## 4. Conclusions

In this work, we rationally designed and synthesized a new fluorescent probe by conjugating the DNB alkylthioether with the **FITA** platform for the first time. The sensing mechanism was verified by spectroscopic studies and HRMS. Thanks to the dual PET effects, the fluorescence of probe is low, and after GSH activation, a >80-fold fluorescence enhancement at 460 nm was observed. Our studies demonstrate that **FITA-FD3** has good sensitivity, appropriate response time, and negligible cytotoxicity. In addition, **FITA-FD3** should be useful for the imaging of endogenous and exogenous GSH in cells. In summary, the DNB alkylthioether is a new biocompatible *S*-protective group that can be triggered by thiols with the assistance of CTAB. This new thiolysis strategy not only further expends the application scope of our **FITA** platform but also extends chemical tools for promoting sulfur-based therapeutic applications. Further work is underway on using similar strategies for the design of new donors, prodrugs, and other fluorescent probes.

## Figures and Tables

**Scheme 2:**
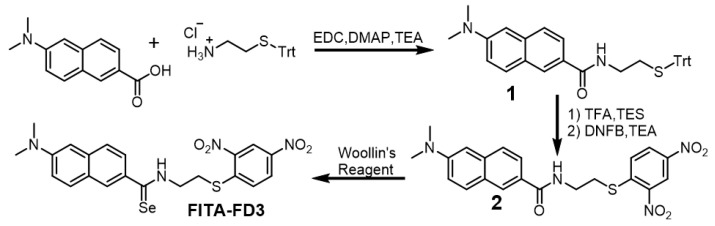
Synthesis of probe **FITA-FD3**.

## Data Availability

Data are available on request from the corresponding author.

## Supplementary Materials

The following supporting information can be downloaded at https://www.mdpi.com/article/10.3390/s25010034/s1: Figure S1: Selected 2,4-dinitrophenyl, 2,4-dinitrobenenesulfonate and 2,4-dinitrobenzensulfonamide-based probes for biothiols; Figure S2: ^1^H NMR (400 MHz, CDCl_3_) of **1**; Figure S3: ^13^C NMR (101 MHz, CDCl_3_) of **1**. Figure S4: HRMS (ESI) of **1**. Figure S5: ^1^H NMR (400 MHz, DMSO-*d*_6_) of **2**. Figure S6: ^13^C NMR (101 MHz, DMSO-*d*_6_) of **2**. Figure S7: HRMS (ESI) of **2**. Figure S8: ^1^H NMR (400 MHz, DMSO-*d*_6_) of **FITA-FD3**. Figure S9: ^13^C NMR (101 MHz, DMSO-*d*_6_) of **FITA-FD3**. Figure S10: ^77^Se NMR (114 MHz, DMSO-*d*_6_) of **FITA-FD3**. Figure S11: HRMS (ESI) of **FITA-FD3**; Figure S12: Spectroscopic confirmation of product **3** from the reaction of **FITA-FD3** and GSH.; Figure S13: Time-dependent absorbance spectra of **FITA-FD3** (40 µM) with 5 mM GSH in PBS (pH 7.4, containing 1 mM CTAB); Figure S14: pH-dependent fluorescence response of **FITA-FD3** (5 μM) with GSH (3 mM) in PBS (50 mM, pH 2.0, 3.0, 4.0, 5.5, 6.5, 7.4, 9.0) for 30 min (a) and 60 min (b) of incubation at 25 °C. Figure S15: HRMS analysis for the coincubation of **FITA-FD3** (0.5 mM) and GSH (5 mM) in PBS buffer (50 mM, pH 7.4, containing 0.3 mM CTAB and 0.5 mM **Cy7-Cl**) overnight at room temperature. The resultant solution was filtrated, and the filtrate was used directly for HRMS tests. Figure S16: Representative time-dependent fluorescence spectra of **FITA-FD3** (5 µM) with 1, 2, or 5 mM GSH (from left to right) in PBS (pH 7.4, containing 1 mM CTAB). Figure S17: Time-dependent emission intensities at 460 nm of **FITA-FD3** (5 μM) with 3 mM biothiols in PBS (pH 7.4) at 25 °C. Figure S18: Relative cell viability of HeLa cells after treatment with probe **FITA-FD3** for 24 h. The results are expressed as mean ± S.D. (*N* = 4).
