# Supplementary material for: FITA-Containing 2,4-Dinitrophenyl Alkylthioether-Based Probe for Detection and Imaging of GSH
Source: Sensors (Basel). 2024 Dec 24;25(1):34. doi: 10.3390/s25010034 (PMC11723303; doi:10.3390/s25010034)
Supplement: Supplementary file 1 [file sensors-25-00034-s001.zip › sensors-3363255-supplementary.pdf]

Supporting Information

FITA-Containing 2,4-Dinitrophenyl Alkylthioether-Based Probe for Detection and Imaging of GSH

Yalun Dong [1], Liyue Wang [2], Wenfang Liang [1], Jiqin Zhu [1,*], Lu Sun [2] and Long Yi [2,*]

1 State Key Laboratory of Organic-Inorganic Composites and Beijing Key Lab of Bioprocess, Beijing University of Chemical Technology, Beijing 100029, China; 2022400048@mail.buct.edu.cn (Y.D.); m13593008045@163.com (W.L.)
2 Tianjin Key Laboratory on Technologies Enabling Development of Clinical Therapeutics and Diagnostics, School of Pharmacy, Tianjin Medical University, Tianjin 300070, China; wly13034198045@163.com (L.W.); sunlu@tmu.edu.cn (L.S.)
* Correspondence: zhujq@mail.buct.edu.cn (J.Z.); yilong@mail.buct.edu.cn (L.Y.)

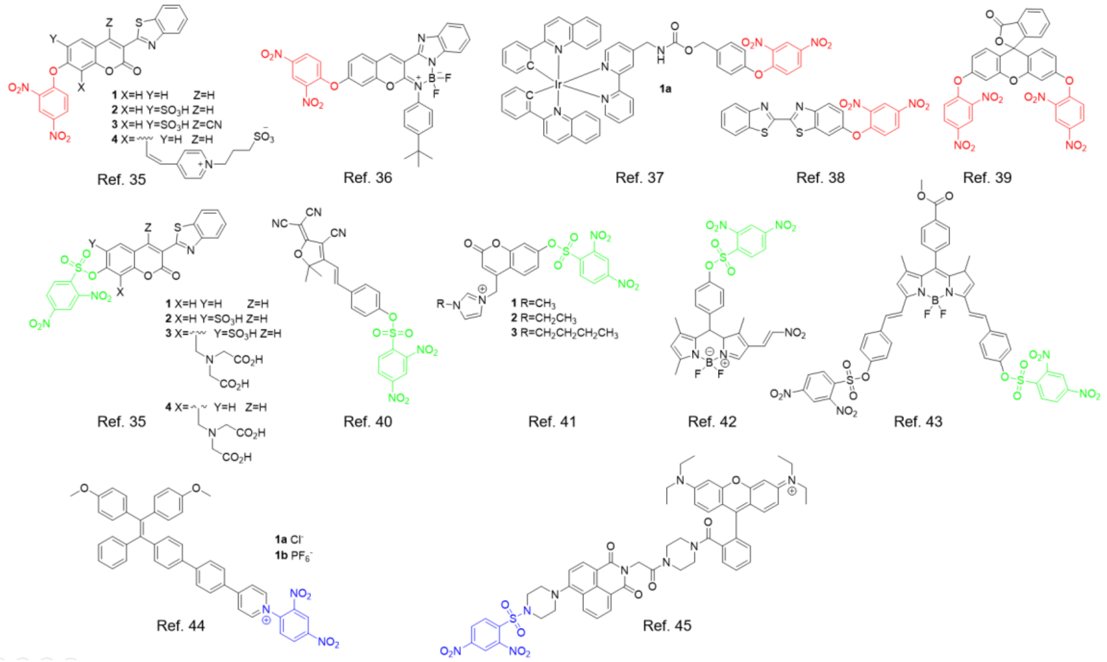

Figure S1. Selected fluorescent probes based on thiolysis of 2,4-dinitrophenyl ethers, 2,4-dinitrophenyl sulfonates, and 2,4-dinitrophenyl sulfonamides for biothiols [35-45].

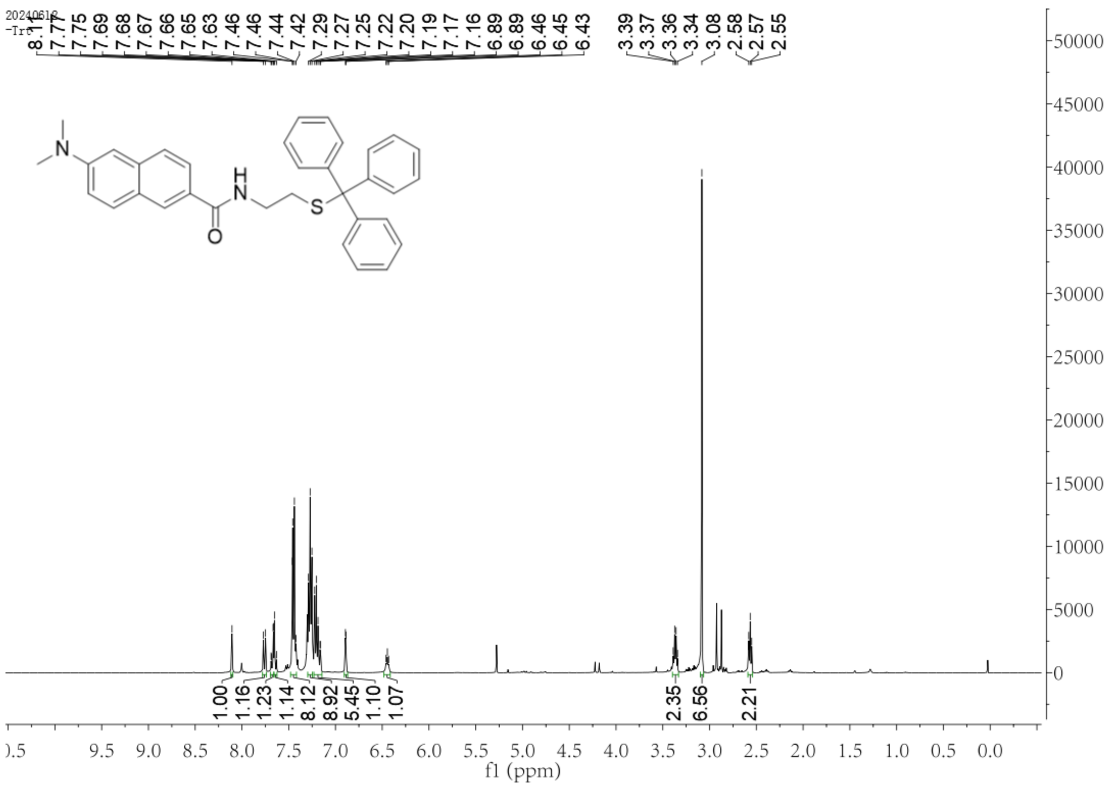

Figure S2. ^{1}H NMR (400 MHz, $CDCl_3$) of **1**.

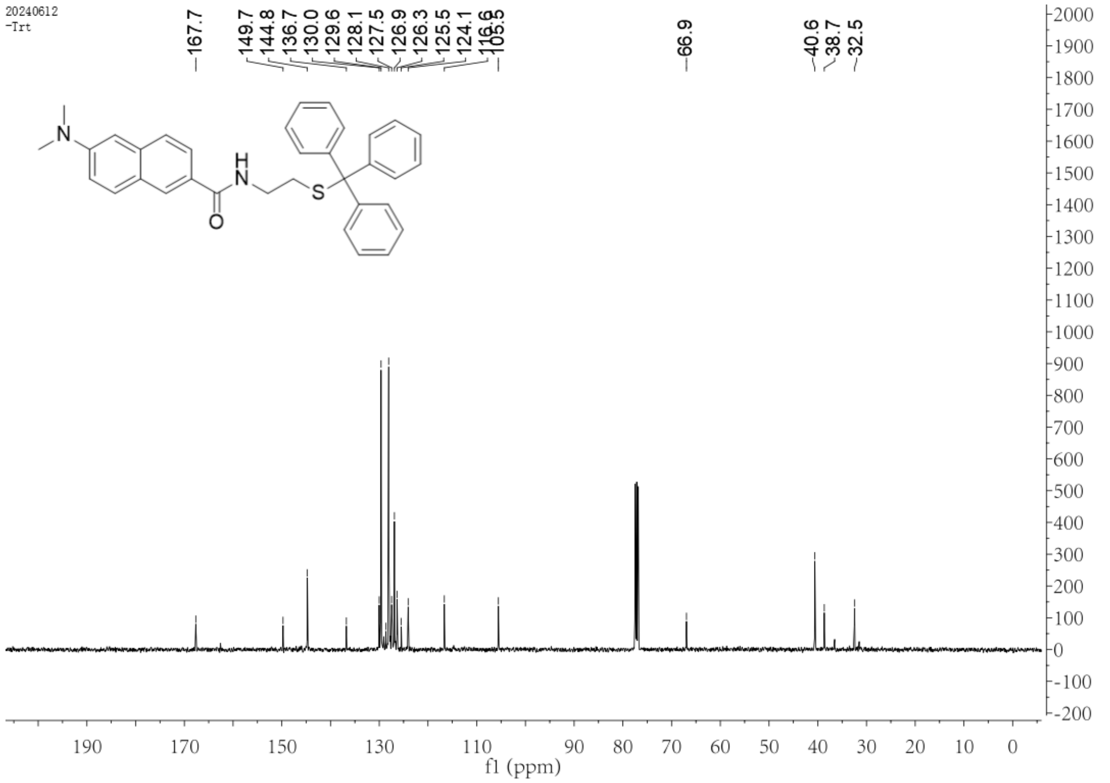

Figure S3. ^{13}C NMR (101 MHz, $CDCl_3$) of **1**.

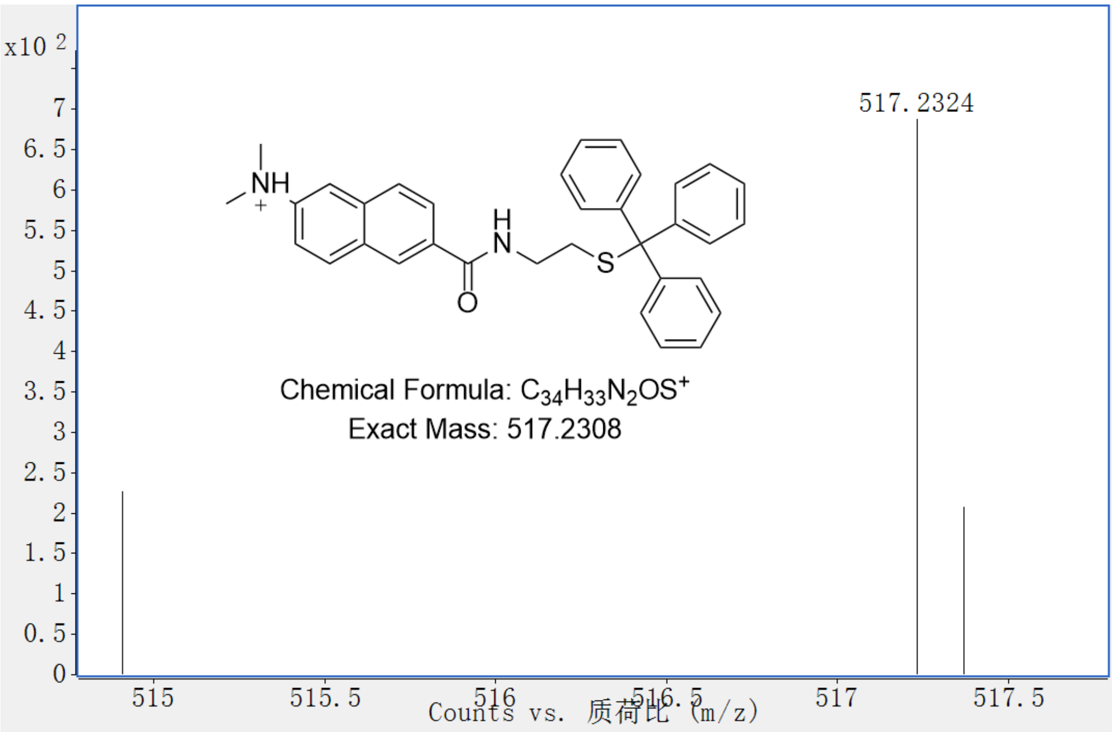

Figure S4. HRMS (ESI) of **1**.

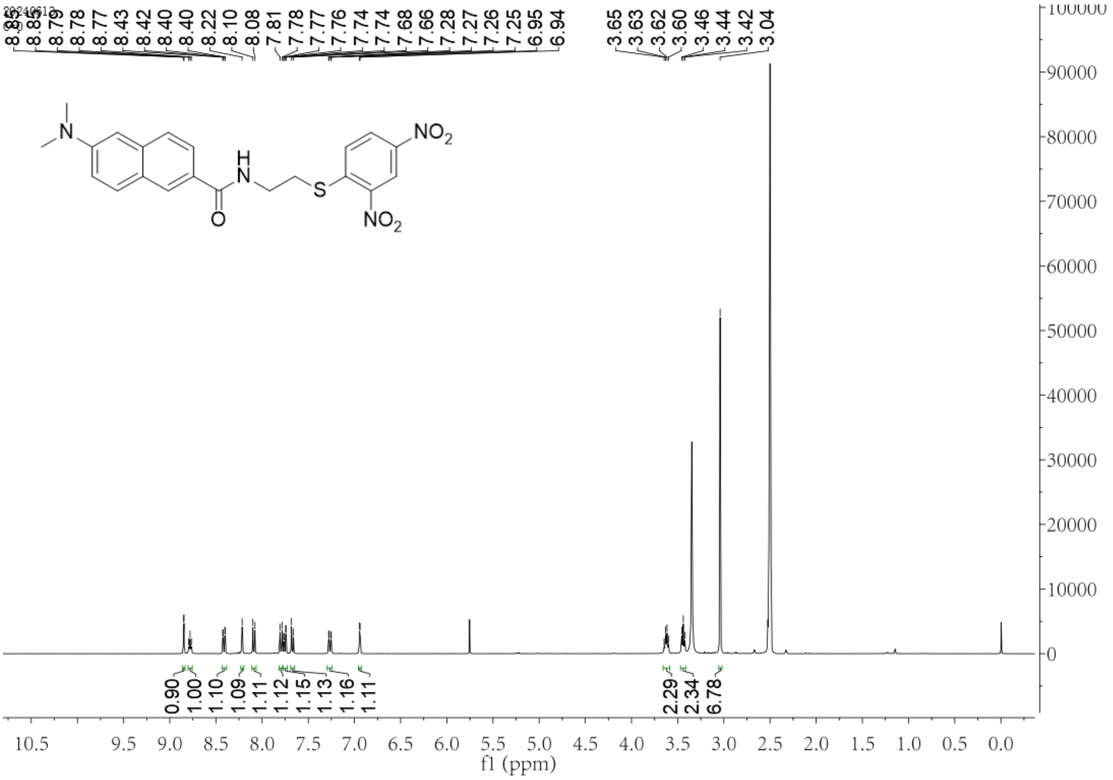

Figure S5. ^{1}H NMR (400 MHz, DMSO-d_6) of **2**.

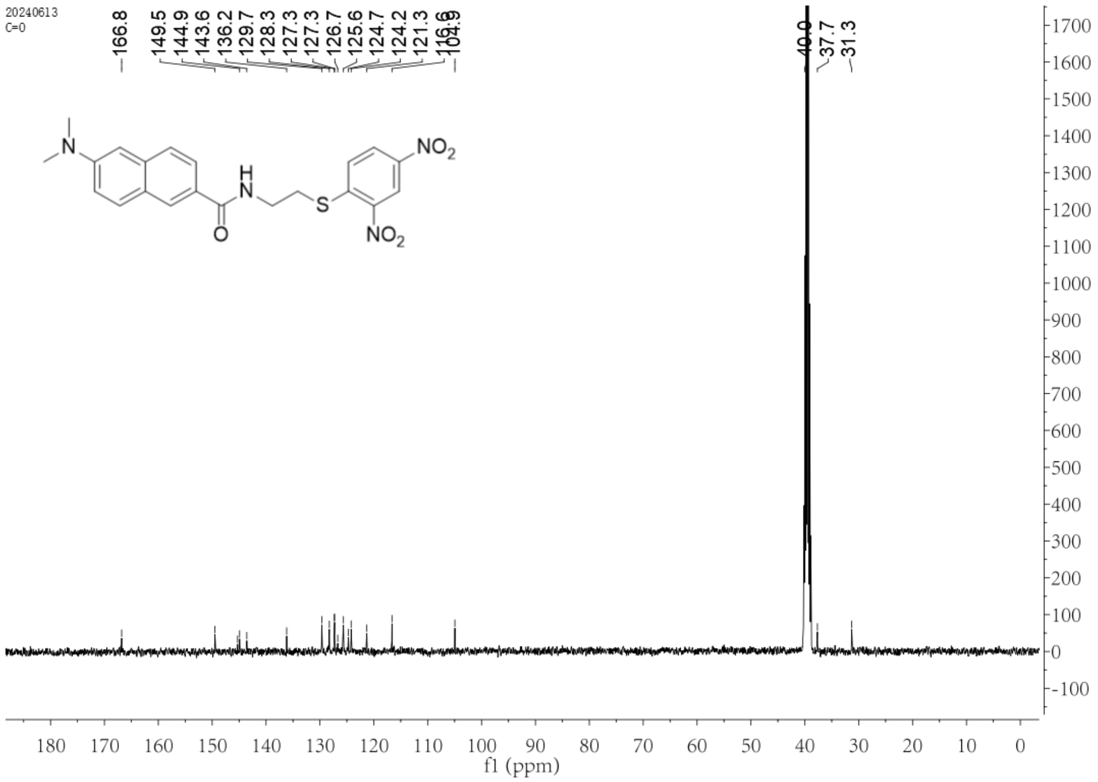

Figure S6. ^{13}C NMR (101 MHz, DMSO-d_6) of **2**.

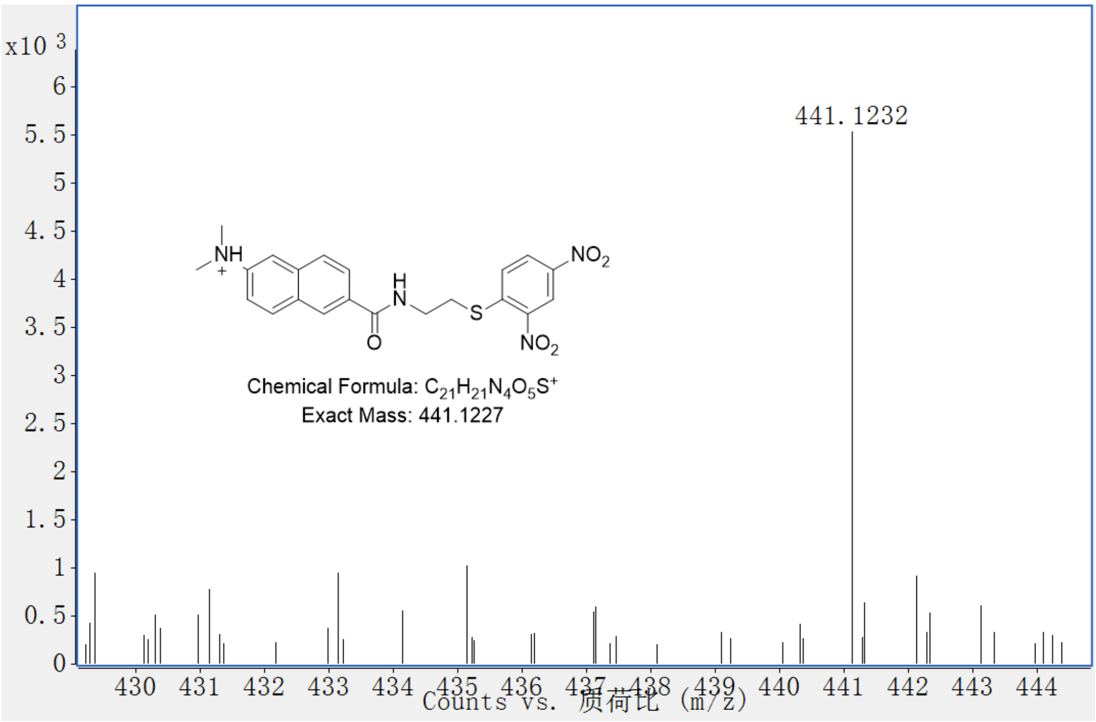

Figure S7. HRMS (ESI) of **2**.

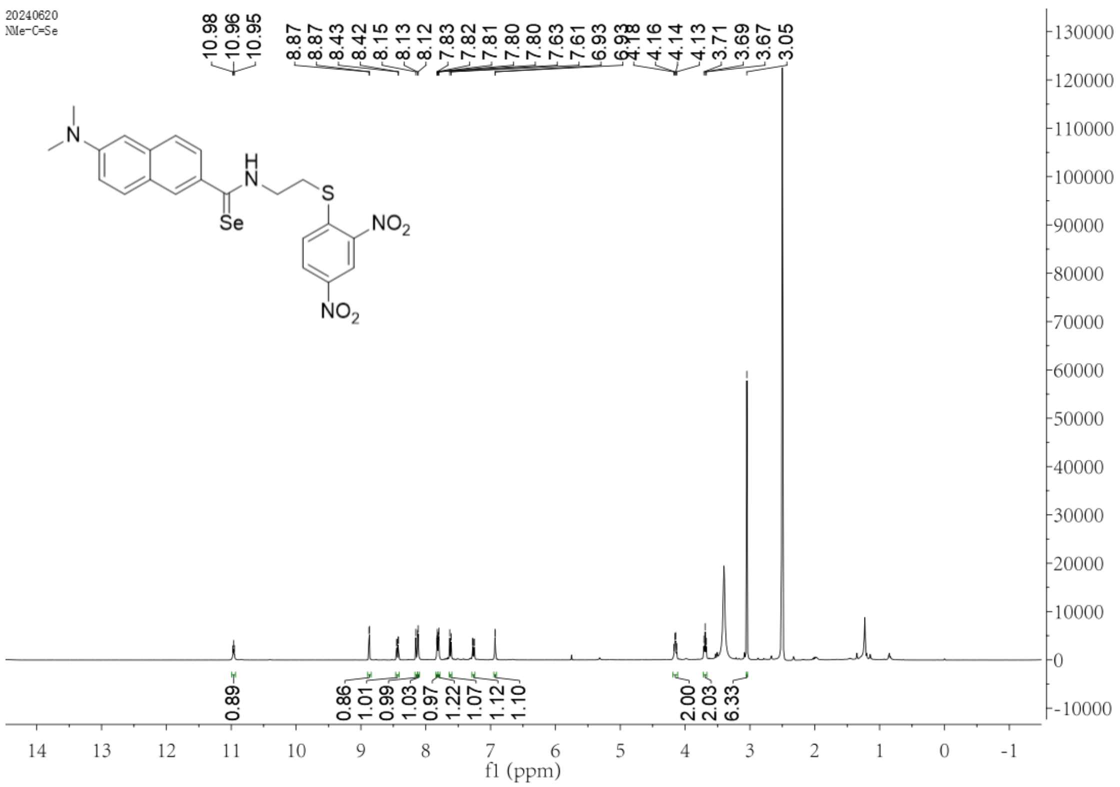

Figure S8. ^{1}H NMR (400 MHz, DMSO-d_6) of **FITA-FD3**.

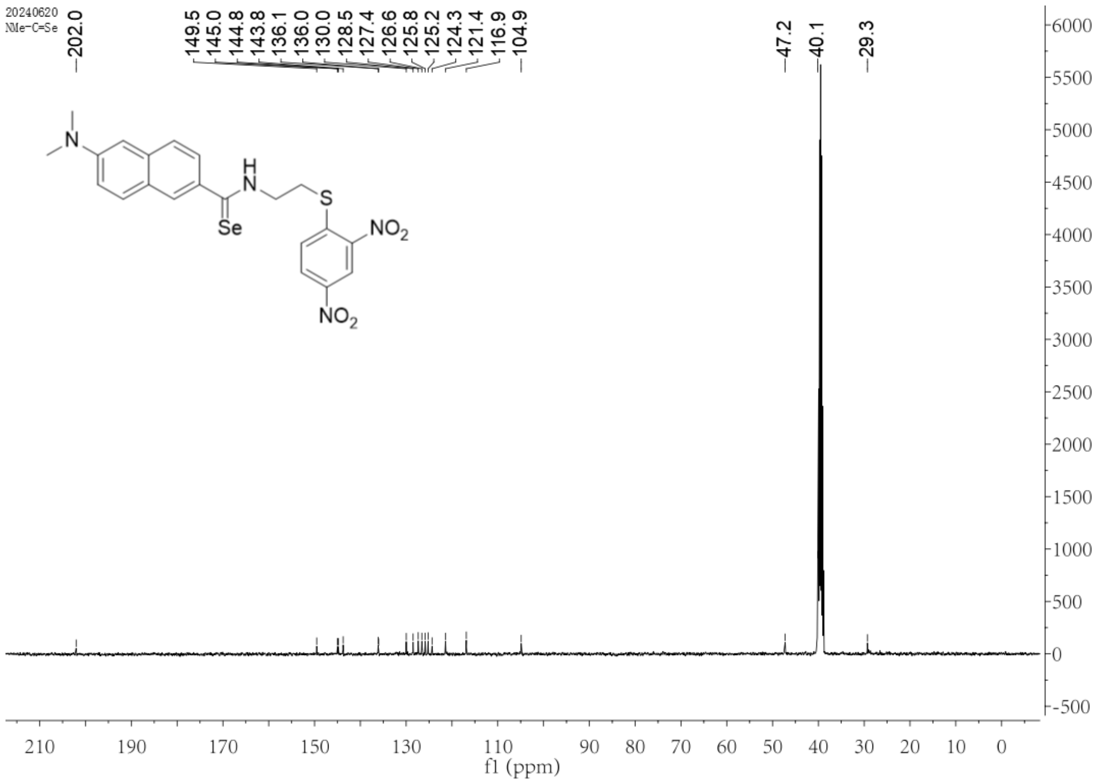

Figure S9. ^{13}C NMR (101 MHz, DMSO-d_6) of **FITA-FD3**.

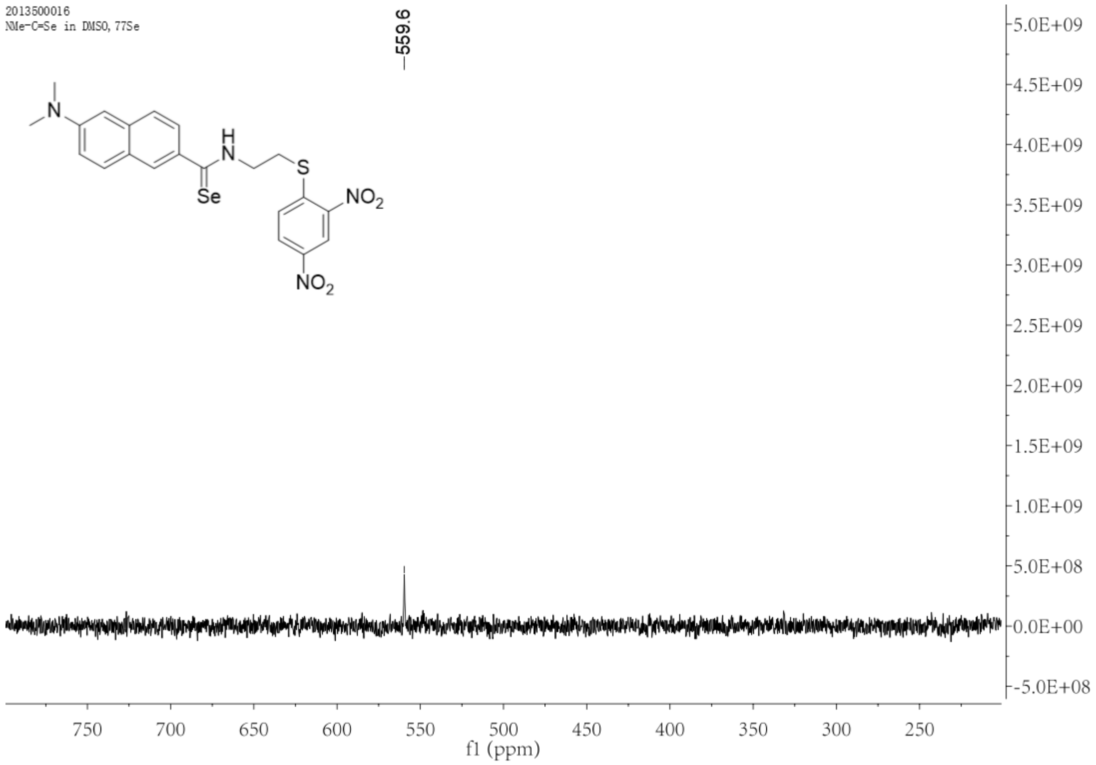

Figure S10. ^{77}Se NMR (114 MHz, DMSO-d_6) of **FITA-FD3**.

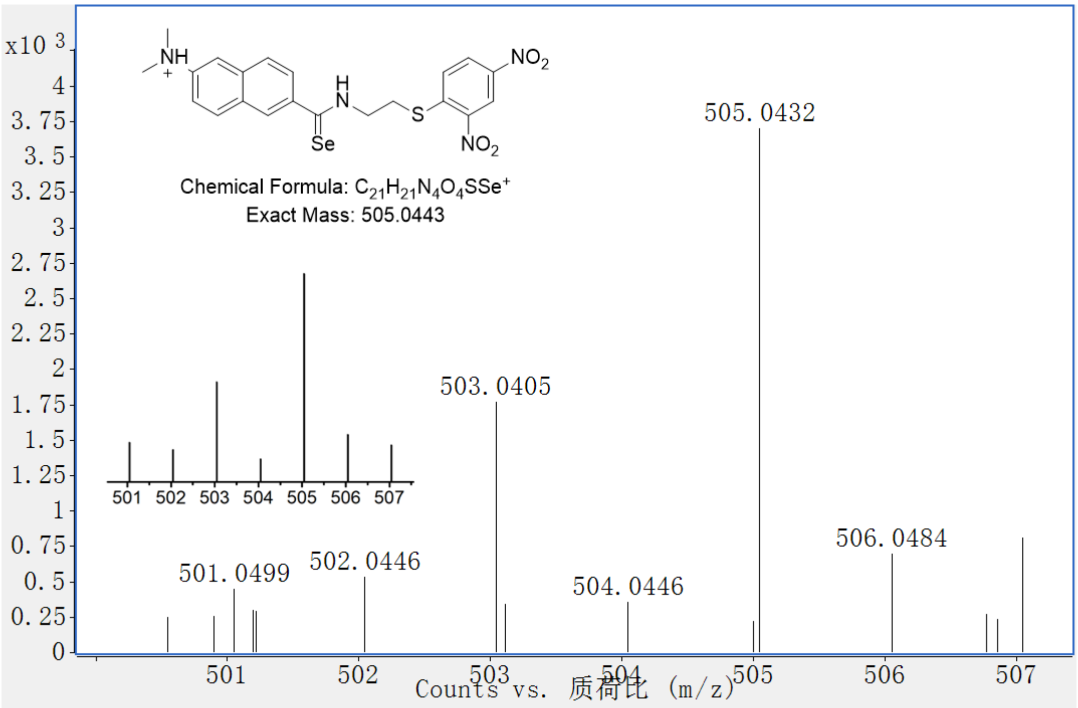

Figure S11. HRMS (ESI) of **FITA-FD3**.

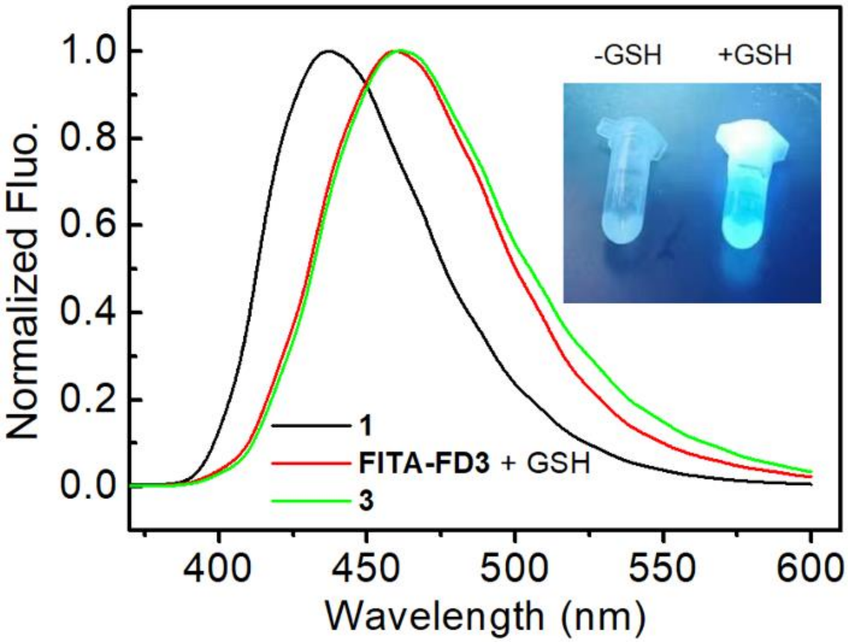

Figure S12. Spectroscopic confirmation of product **3** from the reaction of **FITA-FD3** and GSH. Normalized fluorescence spectra of **1** (5 μM) and the reaction solution of **FITA-FD3** (5 μM) with 5 mM GSH for 30 min in PBS buffer (pH 7.4, containing 1 mM CTAB) and pure **3** (5 μM) with 5 mM GSH in PBS buffer (pH 7.4, containing 1 mM CTAB). Compared with **1**, the emission peak of the reaction solution is significantly red-shifted from 436 nm to 460 nm. The spectrum of the reaction solution overlaps with the spectrum of **3** [48]. Inset: photos under 365 nm irradiation of the solutions of **FITA-FD3** (40 μM) without (left) or with (right) 3 mM GSH for 30 min of incubation.

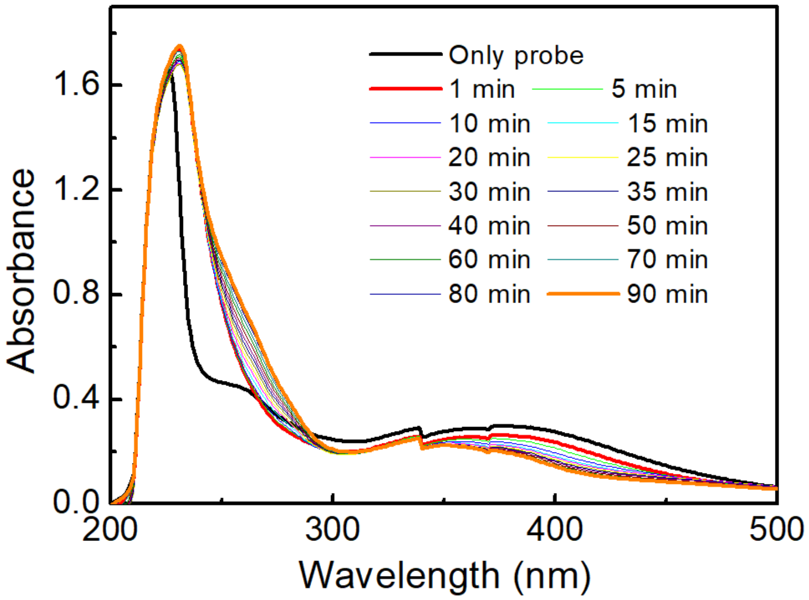

Figure S13. Time-dependent absorbance spectra of **FITA-FD3** (40 μM) with 5 mM GSH in PBS (pH 7.4, containing 1 mM CTAB).

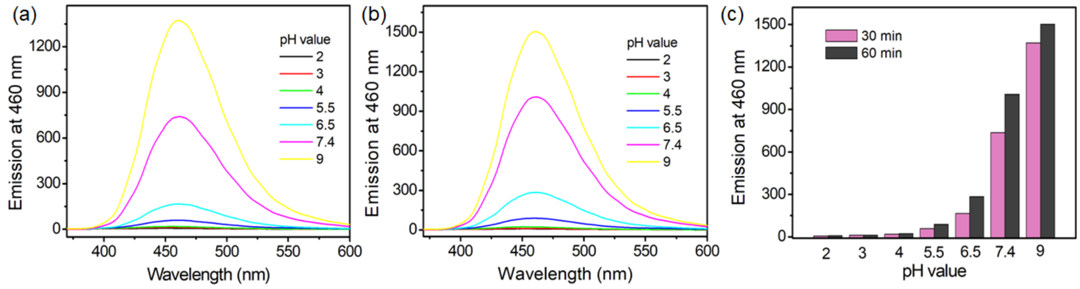

Figure 14. pH-Dependent fluorescence response of **FITA-FD3** (5 μM) with GSH (3 mM) in PBS (50 mM, pH 2.0, 3.0, 4.0, 5.5, 6.5, 7.4, 9.0) for 30 min (**a**) and 60 min (**b**) of incubation at 25 ºC.

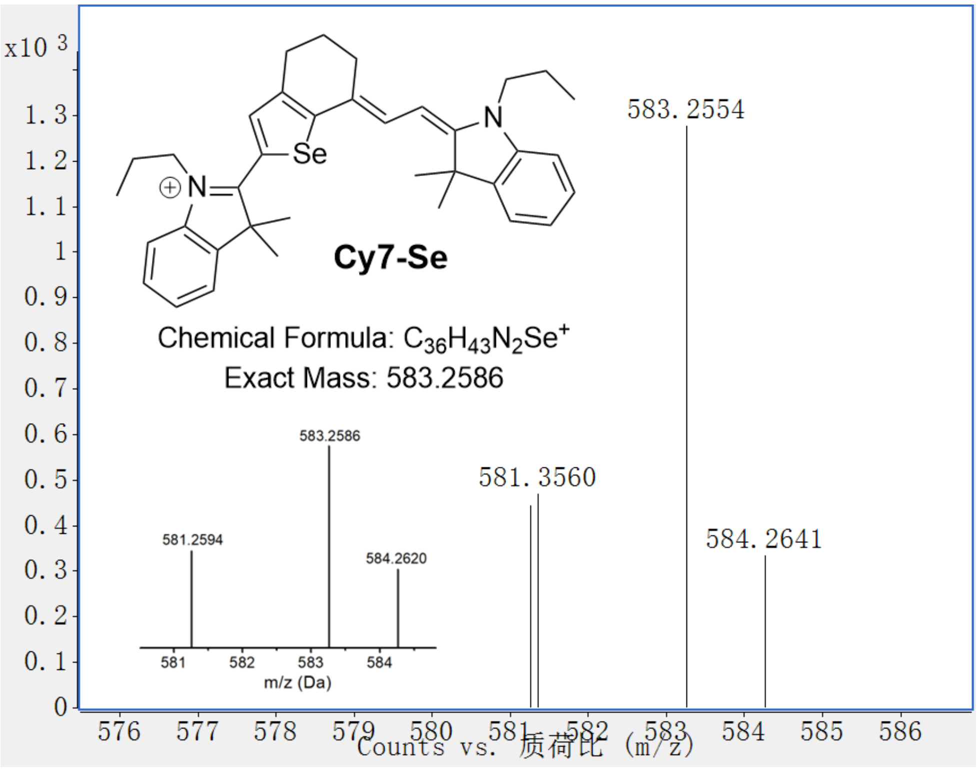

Figure S15. HRMS analysis for the coincubation of **FITA-FD3** (0.5 mM) and GSH (5 mM) in PBS buffer (50 mM, pH 7.4, containing 0.3 mM CTAB and 0.5 mM **Cy7-Cl**) overnight at room temperature. The resultant solution was filtrated, and the filtrate was used directly for HRMS tests.

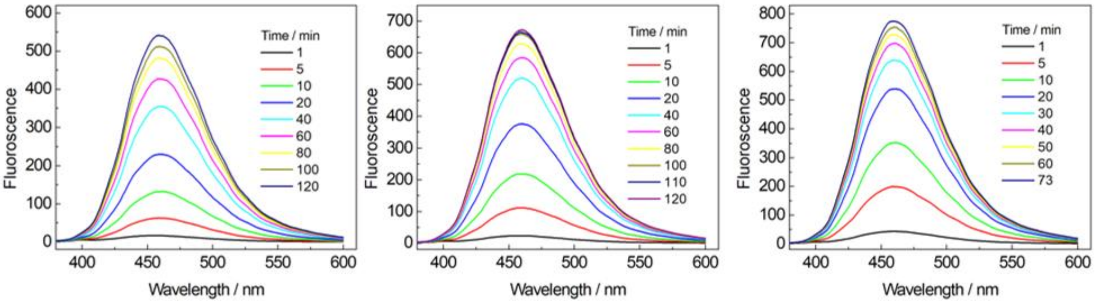

Figure S16. Representative time-dependent fluorescence spectra of **FITA-FD3** (5 μM) with 1, 2, or 5 mM GSH (from left to right) in PBS (pH 7.4, containing 1 mM CTAB).

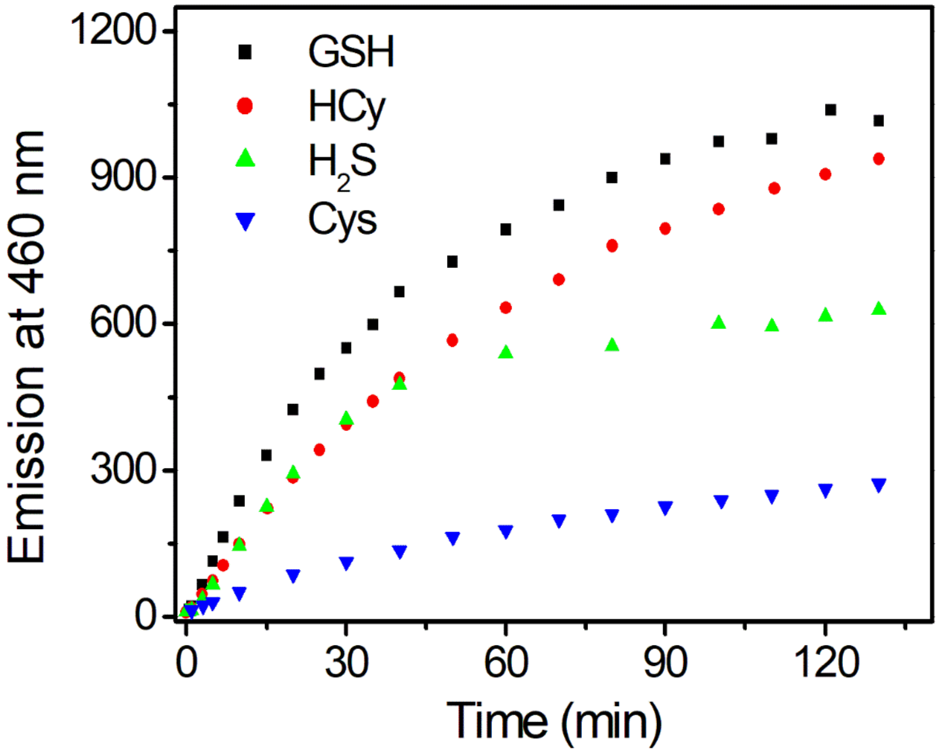

Figure 17. Time-dependent emission intensities at 460 nm of **FITA-FD3** (5 μM) with 3 mM biothiols in PBS (50 mM, pH 7.4) at 25 °C.

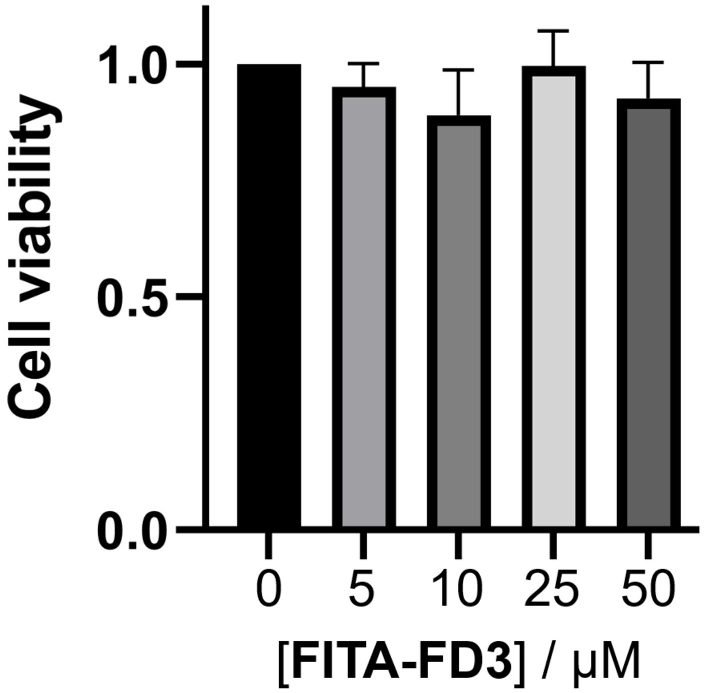

Figure S18. Relative cell viability of HeLa cells after treatment with probe **FITA-FD3** for 24 h. The results are expressed as mean ± S.D. (n = 4).
